# Effects of Taraxerol on Oxidative and Inflammatory Mediators in Isoproterenol-Induced Cardiotoxicity in an Animal Model

**DOI:** 10.3390/molecules28104089

**Published:** 2023-05-15

**Authors:** Alhussain H. Aodah, Sushma Devi, Faisal K. Alkholifi, Hasan S. Yusufoglu, Ahmed I. Foudah, Aftab Alam

**Affiliations:** 1 Department of Pharmaceutics, College of Pharmacy, Prince Sattam Bin Abdulaziz University, Al Kharj 11942, Saudi Arabia; a.aodah@psau.edu.sa; 2 Chitkara College of Pharmacy, Chitkara University, Rajpura 140401, Punjab, India; devi.sushma@chitkara.edu.in; 3 Department of Pharmacology & Toxicology, College of Pharmacy, Prince Sattam Bin Abdulaziz University, Al Kharj 11942, Saudi Arabia; 4 Department of Pharmacognosy & Pharmaceutical Chemistry, College of Dentistry & Pharmacy, Buraydah Private Colleges, Buraydah 51418, Saudi Arabia; hasan.yusuf@bpc.edu.sa; 5 Department of Pharmacognosy, College of Pharmacy, Prince Sattam Bin Abdulaziz University, Al Kharj 11942, Saudi Arabia; a.foudah@psau.edu.sa

**Keywords:** isoproterenol, taraxerol, myocardial infarction, lactate dehydrogenase, creatine kinase, superoxide dismutase, glutathione peroxidase, TNF-α, interleukins

## Abstract

Myocardial infarction (MI) continues to be an important issue in healthcare systems worldwide, leading to high rates of morbidity and mortality. Despite ongoing efforts towards the development of preventive measures and treatments, addressing the challenges posed by MI remains difficult both in developed and developing countries. However, researchers recently investigated the potential cardioprotective effects of taraxerol utilizing an isoproterenol (ISO)-induced cardiotoxicity model among Sprague Dawley rats. Specifically, subcutaneous tissue injections consisting of 5.25 mg/kg or 8.5 mg/kg ISO were administered over two consecutive days as stimuli to induce cardiac injury. To investigate the possibility of preventing damage caused by ISO-induced cardiotoxicity by taraxerol treatment, five groups were formed: a normal control group (1% Tween 80), an ISO control group, an amlodipine group administered 5 mg/kg/day, and various doses of taraxerol. The study results showed that treatment significantly reduced cardiac marker enzymes. Additionally, pretreatment with taraxerol increased myocardial activity in SOD and GPx, leading to significant reductions in serum CK-MB levels along with MDA, TNF-α, and IL-6. Further histopathological analysis supported these observations, as treated animals had less cellular infiltration compared to untreated ones. These multifaceted findings suggest that oral administration of taraxerol could potentially protect hearts from ISO-caused damage by increasing endogenous antioxidant concentrations while decreasing pro-inflammatory cytokines.

## 1. Introduction

Cardiovascular diseases, also known as CVD, encompass a variety of medical conditions that have significant implications for public health worldwide. These complex ailments impact both the heart and blood vessels and are an increasing concern for healthcare professionals globally [1]. Among this broad category of illnesses lies several specific yet interrelated conditions including but not limited to atherosclerosis, stroke, coronary artery disease (CAD), peripheral artery disease (PAD), hypertension or high blood pressure, heart failure, and various cardiac arrhythmias; each with their own set of unique challenges requiring specialized care from clinicians [2]. The increasing occurrence of CVD requires individuals worldwide to prioritize prevention through physical activity, healthy eating habits, and risk factor management, such as quitting smoking or maintaining a safe weight. This promotes a longer and healthier life free from these debilitating conditions [3].

Severe cardiovascular diseases are a significant and increasing concern in the United States, ranking as one of the primary causes of death. Recent statistics show that CVD results in more annual fatalities than any other cause. Urgent attention is required to raise awareness about risk factors and preventive measures to combat these debilitating ailments’ prevalence rate [4,5]. Myocardial infarction (MI) is a serious medical condition that can cause irreversible damage to the heart and even lead to fatalities. Recent research suggests that 75% of sudden cardiac deaths are associated with prior MI, making it one of modern medicine’s critical issues with up to a 5% increased risk [6]. The data underscores the critical importance of prompt medical care and preventive measures for individuals with a history of cardiac problems, particularly those who have suffered from myocardial infarction. Healthcare providers need to consistently monitor and address underlying cardiovascular issues in order to decrease risk factors and enhance overall prognosis [7].

MI occurs when the amount of blood entering the coronary arteries is insufficient to meet the demands of the heart muscle, resulting in tissue destruction [8]. The extent of damage caused by MI correlates directly with factors such as inflammatory responses, the production of damage-associated molecular patterns, the concentration of reactive oxygen species (ROS), and the release of multiple cytokines that regulate immune cell migration toward injury sites. Cytokines play a crucial role in controlling and directing this flow toward affected areas. These intricate pathways involving various mediators contribute significantly to post-MI tissue repair processes at both local and systemic levels through their orchestration [9,10]. The profibrotic pathway is a complex process that results in scarring and remodeling of the ventricular wall due to sustained pathogenic abnormalities. These adaptations showcase how cardiac tissue adapts under stress, with multifaceted processes interplaying for adequate compensation against ongoing stressors. The body’s remarkable ability to adapt towards optimal function even in challenging circumstances is demonstrated through these mechanisms [11].

MI diseases are becoming increasingly common and dangerous due to their progressive nature. Cardiology research has made great strides in recent years, seeking drugs that can protect against these ailments. Preventative treatment with beta-blockers, calcium antagonists, angiotensin-converting enzymes, and antioxidants shows promise but often comes with side effects or high dosages. This highlights the need for alternative therapies that offer better efficacy without compromising safety through further research [6,12]. Researchers globally are increasingly studying novel cardioprotective drugs in response to the growing incidence of cardiovascular diseases. Active plant-based compounds have gained significant attention as potential remedies for severe medical conditions due to their natural origins and perceived efficacy without adverse reactions commonly found in synthetic medications. Despite notable progress, further research is needed to fully comprehend these medicinal properties’ biological mechanisms and develop practical treatment options applicable to diverse populations [13]. The market for herbal medicines is predicted to grow and reach USD 111 billion by the end of 2023. Various studies suggest that these remedies constitute a considerable proportion of pharmaceutical phytoconstituents used in clinical practice [14].

The inflammatory response plays a vital role in myocardial injury, repair, and remodeling after MI, according to different researchers. Furthermore, experimental studies have demonstrated the impact of inflammation on heart health. To address an overactive response, anti-inflammatory chemicals can be promoted; taraxerol is one such natural compound found in higher plants belonging to the Asteraceae family that has potential therapeutic benefits due to its pentacyclic triterpenoid structure lacking a methyl group at position 14 but possessing α-methyl substituent at position 13 and containing double bonds between positions 14 and 15. Incorporating taraxerol into treatments may effectively reduce inflammation within cardiac tissues [15]. Taraxerol has some reported pharmacological effects, including antitumor [16], antioxidant [17], anti-microbial [18], anti-inflammatory [19,20,21], antidiabetic [22,23], and anti-Alzheimer [23]. Taraxerol is a triterpenoid compound shown to have powerful anti-inflammatory properties [24]. The anti-inflammatory properties of taraxerol have been observed by several researchers. A study by Yao et al. found that taraxerol hinders NF-κB activation, leading to diminished pro-inflammatory mediator generation in macrophages. Taraxerol accomplishes this feat via interference with TAK1 and Akt activation, thereby halting the process of NF-κB stimulation [20]. PI3K and Akt act as connection molecules that link extracellular signals with cellular reactions, and they play a significant role in regulating the NF-κB pathway [25].

A thorough peer review has determined that although many studies have demonstrated taraxerol’s potential positive impact on various diseases, its efficacy in treating inflammatory conditions is still uncertain. However, our latest research concentrates on comprehending the therapeutic mechanism of taraxerol as a treatment for MI induced by Isoproterenol (ISO) in rats. It should be noted that ISO—acting as both a synthetic catecholamine and adrenergic agonist—can lead to infarct-like necrosis when administered at high doses [26]. In experimental animals, it has been noted that high catecholamine concentrations (isoproterenol) might result in myocardial infarction by causing necrotic lesions in the heart [27]. The pathophysiological and morphological changes in rat cardiac muscle after ISO administration are comparable to those observed during human MI. Consequently, this model has emerged as a widely accepted standard for examining heart functions and evaluating the therapeutic impact of various medications [28]. The study investigates if taraxerol can protect and repair the heart in rats by examining its impact on immunological dysregulation and inflammation.

## 2. Results

### 2.1. Effect of Taraxerol Pretreatment on Heart Rate, Heart Weight, and Blood Pressure

**Figure 1:**
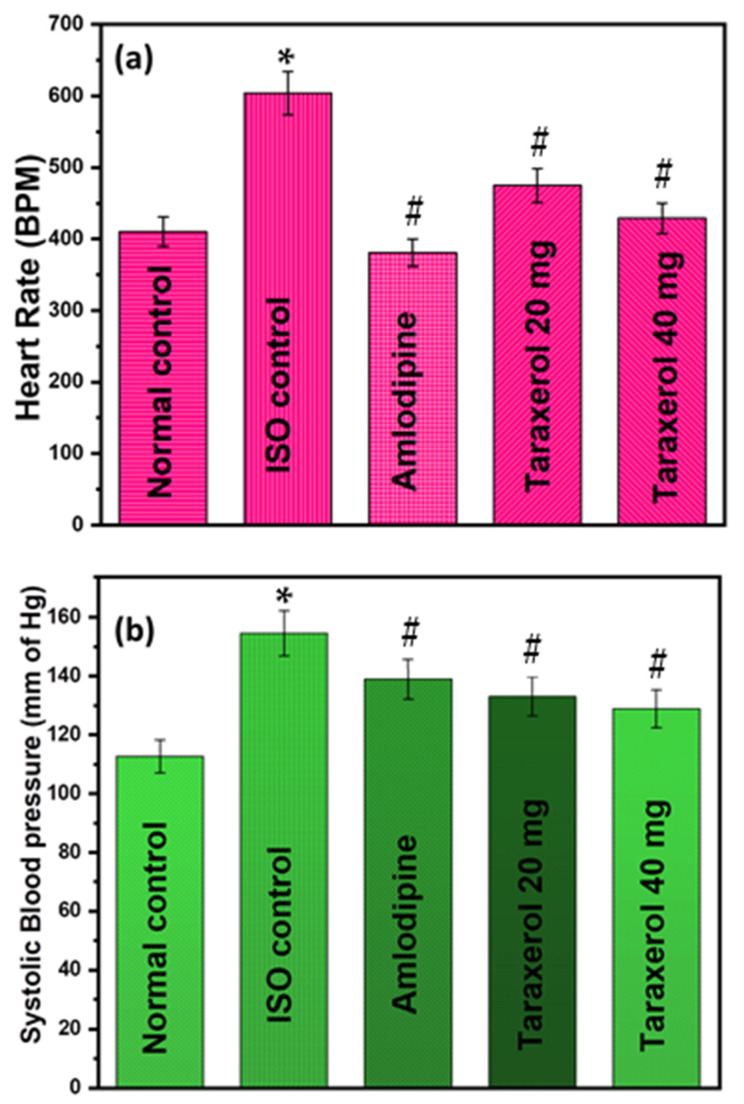
Effect of taraxerol pretreatment on (**a**) heart rate; and (**b**) blood pressure. * *p* < 0.01, as compared with normal control; # *p* < 0.01, as compared with ISO control.

The study observed no significant changes in terms of heart or overall body weight among different groups of rats during the experiment. However, it is worth mentioning that compared to other groups; the ISO control group showed a notable increase in both heart rate and systolic blood pressure parameters (*p* < 0.01). On the other hand, administering taraxerol as pretreatment before subjecting them to only ISO treatment resulted in significant reductions in both heart rate and systolic blood pressure (*p* < 0.01) as shown in Figure 1. These findings suggest that using taraxerol prior to exposure to stressors like isoproterenol may prove useful for tackling cardiac ailments similar to hypertension-like symptoms displayed by rodents during experimental conditions, particularly at doses higher than 40 mg/kg owing to its dose-dependent effect found through experimentation.

### 2.2. Effect of Taraxerol Pretreatment on Cardiac Biomarker Levels

**Figure 2:**
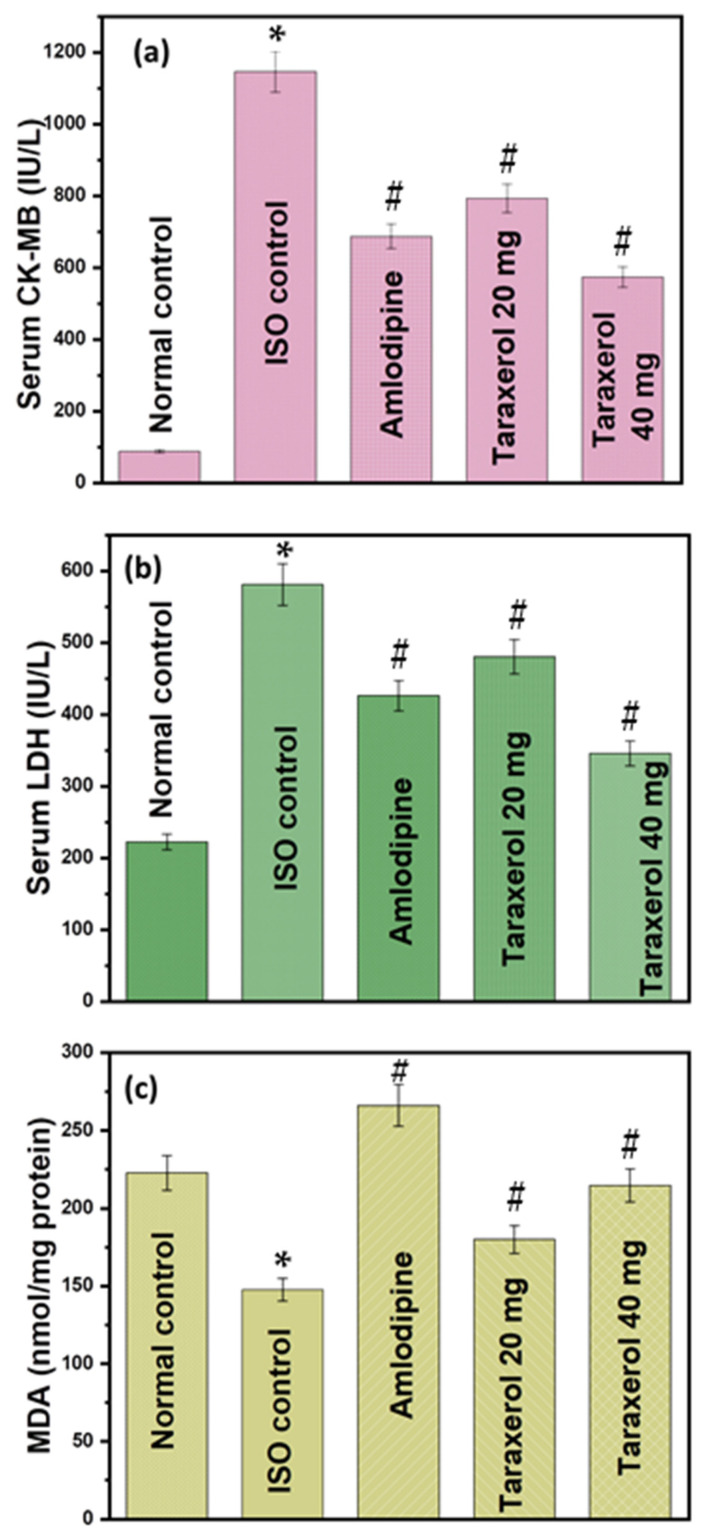
Effect of Taraxerol pretreatment on cardiac biomarker levels (**a**) Serum CK-MB (IU/L); (**b**) Serum LDH (IU/L); (**c**) MDA (nmol/mg protein) * *p* < 0.01, as compared with normal control; # *p* < 0.01, as compared with ISO control.

The results presented in Figure 2 show a remarkable increase in the activities of the marker enzymes for myocardial damage in serum, namely CK-MB and LDH, after treatment with ISO compared with their values in control rats. This observation suggests that the administration of ISO resulted in significant damage to the myocardium, as reflected by an increase in these enzyme markers, which are commonly used as indicators for detecting cardiac problems. Taraxerol pretreatment at doses of 20 and 40 mg/kg helped to restore isoproterenol-induced changes in serum diagnostic enzymes to normal levels. In addition, taraxerol resulted in a significant decrease in MDA levels after isoproterenol administration. Taraxerol also counteracted the deleterious effect of ISO by significantly increasing MDA levels in a dose-dependent manner (*p* < 0.01).

### 2.3. Effect of Taraxerol Pretreatment on Antioxidant Levels

The study showed a remarkable change in the rats exposed to ISO control, where an increase in MDA levels and a decrease in SOD and GPx activities were observed in their serum. However, administration of taraxerol before this exposure resulted in significant changes, as evidenced by decreased MDA levels and increased antioxidant activity of the enzymes SOD and GPx (as shown in Table 1). These results provide valuable insight into the potential protective effects of taraxerol against oxidative stress induced by the control of ISO.

### 2.4. Effect of Taraxerol Pretreatment on Inflammatory Mediator Levels in the Serum

**Figure 3:**
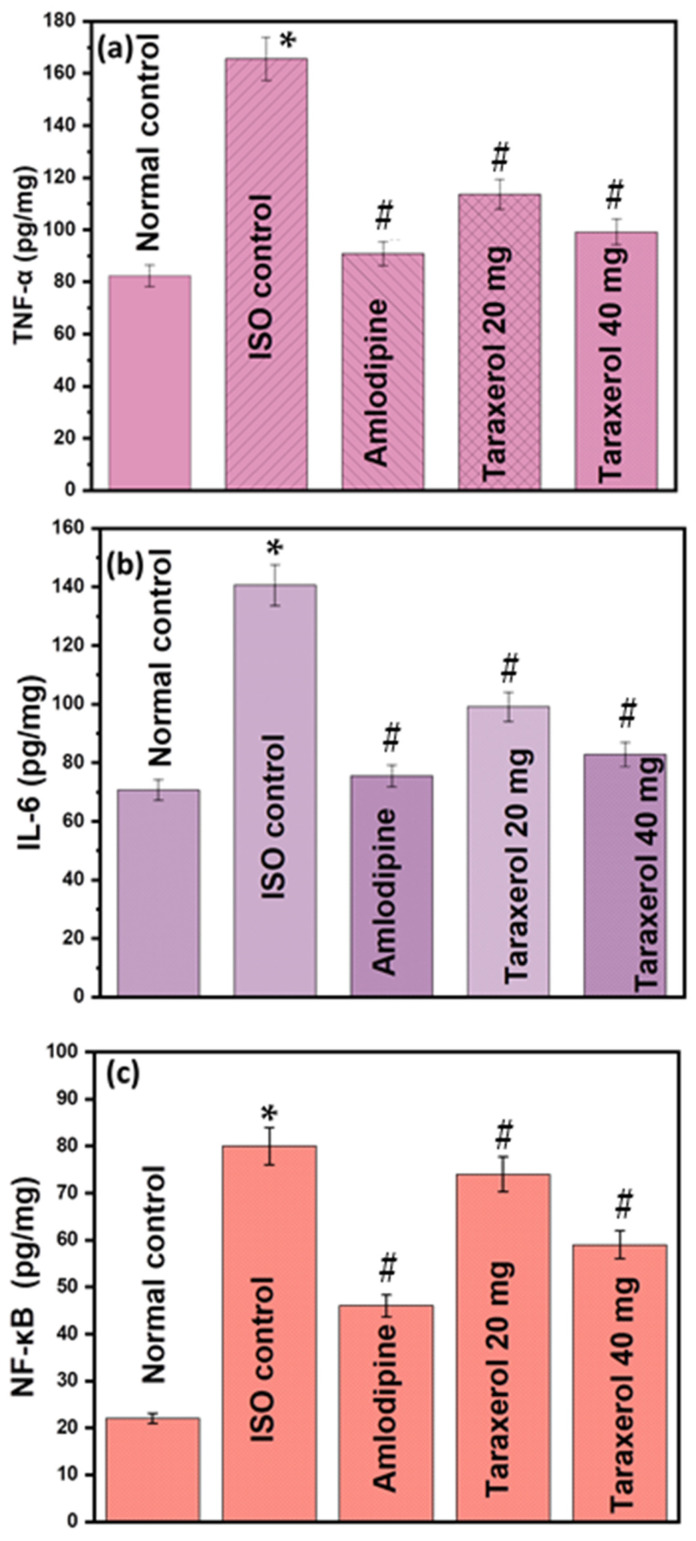
Effect of Taraxerol pretreatment on (**a**) TNF-α, (**b**) IL-6, and (**c**) NF-kB. Data are given as means ± SEM (n = 6). * *p* < 0.01, as compared with normal control; # *p* < 0.01, as compared with ISO control.

The study demonstrated that rats treated with ISO exhibited a significant increase in serum levels of TNF-α and IL-6 as compared to their control counterparts. However, pretreatment with taraxerol resulted in a considerable reduction of these two cytokines among those animals treated with ISO. Notably, no observable changes were noticed within the typical control cohort (Figure 3). These findings imply that taraxerol possesses potential anti-inflammatory characteristics by curbing pro-inflammatory responses linked to heightened TNF-α and IL-6 levels. Please review this accordingly.

### 2.5. Effect of Taraxerol Pretreatment on Heart Histology 

**Figure 4:**
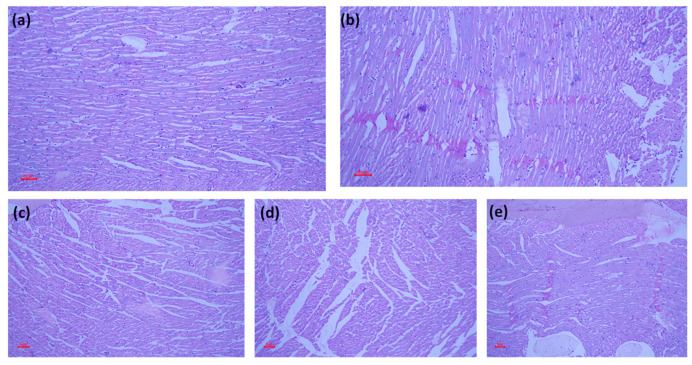
Heart histology of experimental animals from different groups: (**a**) Normal; (**b**) ISO; (**c**) Amlodipine; (**d**) Taraxerol 20 mg/kg; (**e**) Taraxerol 40 mg/kg.

A light micrograph of a control rat’s heart reveals its typical structure. This study reveals the effects of ISO and taraxerol administration on rat heart tissue. First, the structure of control rat hearts was observed, followed by examination after administering ISO to induce damage. This resulted in myophagocytosis, local necrosis, inflammation infiltration, edema, and fibroblastic proliferation. Comparing these results with different doses (20 mg/kg vs. 40 mg/kg) of taraxerol showed significant improvements in myocardial damage, such as less inflammation, myonecrosis, and edema. It should be noted that despite any challenges or injuries sustained during experiments, all subjects studied maintained their typical cardiac fiber configuration, as shown in Figure 4.

## 3. Discussion

Isoproterenol is a synthetic catecholamine, and catecholamines are known to be the culprits behind the structural, functional, and metabolic changes and the necrosis that can occur in rat heart muscle. An ISO-induced myocardial infarction is a well-standardized model that may be used to investigate the protective effects of various medications and how the heart works [29]. In rats, MI is caused by a disruption in the normal balance between the creation of free radicals and the body’s antioxidative defense system [30]. Isoproterenol led to the development of cardiac lesions in rats that resembled infarcts. Several medicines, such as calcium antagonists or sympatholytics, can completely or partially prevent the development of these lesions [30,31]. In the current study, we found a decrease in the activities of myocyte damage markers called CK and LDH in the hearts of rats given ISO. When myocardial cells are injured due to a lack of oxygen or glucose, the integrity of the cell membrane is disrupted, and the cell membrane may become more porous; this leads to the enzymes leaking out [32]. Taraxerol, administered orally before treatment, was able to restore the activity of cardiac marker enzymes. This could be because taraxerol has a protective effect on the myocardium, minimizing the amount of damage to the myocardium and, as a result, limiting the amount of CK and LDH that escapes into the bloodstream.

In the pathology of myocardial necrosis, free radicals, and lipid peroxidation are generated. Our study compared the level of MDA in normal control and ISO control and found that there was a rise in MDA levels. Taraxerol pretreatment lowered the MDA levels in rats, which can occur due to the antioxidant potential of taraxerol. Many studies have reported the antioxidant ability of taraxerol [33,34,35]. It is also possible that the increased activity of antioxidant enzymes (SOD and GPx) is responsible for the lower MDA levels [36]. MDA is also a marker for lipid peroxidation and protein quantification [37,38]. Our findings are consistent with those in earlier investigations [25,36].

It has been established that oxidative stress is a critical factor in the pathogenesis of myocardial infarction and other cardiac diseases. Oxygen radicals can potentially cause detrimental effects on the heart, such as malfunction in the contractile and structural damage. According to our findings, rats with ISO-induced myocardial infarction had dramatically increased oxidant activities in their serum, and taraxerol attenuated this increment [39]. Compared with the other groups, the SOD activity was lower in the group that received the administration of ISO. During an MI, superoxide radicals are formed at the site of damage, which modifies SOD, resulting in loss of activity and accumulation of superoxide radicals, both of which damage the myocardium [37]. These results mimic the prior results [40,41]. Taraxerol pretreatment restored the decreased activity of SOD, which indicated improved superoxide radical elimination.

This study found that the administration of ISO decreased the activity of the GPx enzyme found in the heart of the animals [42]. A decrease in the activity of GPx can potentially lead to a buildup of hazardous chemicals as a consequence of oxidative damage [43]. However, after treatment with taraxerol, the decreased activity of GPx was restored, which provides evidence of the antioxidant capacity of the triterpenoid molecule and its potential function in providing protection.

Hemorrhages in the myocardium and epicardium, in addition to well-defined lesions, were observed under the microscope during the histopathological examination of heart sections taken from rats treated with ISO. These lesions were found most prominently in the apical region of the heart. ISO causes lesions in animals similar to those caused by myocardial infarction by acting on adrenergic receptors. These receptors stimulate intracellular calcium influx, which raises cAMP levels and depletes high-energy phosphates [44,45]. These lesions have a morphological appearance comparable to myofibrillar degeneration, cardiac enlargement, myocyte destruction, and cardiomyopathy [46], including MI. The rats pretreated with taraxerol exhibited normal myofibrillar architecture, including striations, a branching appearance, and continuity with the myofibrils close to them. Taraxerol could defend against and effectively prevent the harmful effects caused by the therapy with ISO.

Numerous synthetic medications were utilized as anti-inflammatory agents to lessen myocardial damage since inflammatory responses and signals play a significant part in cardiovascular disease (CVD). According to a recent study, excessive levels of the proinflammatory cytokines TNF-α and IL-6 cause myocardial damage, which is primarily caused by the activation of NF-kB [47,48]. NF-kB is typically stopped from activating the inhibitory kappa B (IkB) family. The IkB kinase enzymes phosphorylate and break down IkB under MI circumstances. As a result, IkB is unable to inhibit NF-kB, which is then given the green light to function and translocate from the cytosol to the nucleus, bind to the promoter sequence of target genes, and trigger the transcription of proinflammatory cytokines like TNF-a and IL-6 [47]. In the current investigation, ISO-induced mice showed significantly higher TNF-α, IL-6, and NF-kB levels. Our findings showed that, compared to control rats, pre-treatment with taraxerol reduced levels of TNF-α, IL-6, and NF-kB in the heart, indicating that it has anti-inflammatory effects that safeguard the heart tissue.

Taraxerol can be found in plants of the Asteraceae family and the subfamilies Euphorbiaceae and Malvaceae. It has been observed that the majority of members of the Euphorbia genus have demonstrated a significant buildup of taraxerol [15]. Antioxidants can be found in abundance among the plants that belong to this family. Additionally, when used as an anti-inflammatory medication, taraxerol exhibits the most potent pharmacological characteristics. This is one of its functional features. According to Yao et al. and co-workers [47], taraxerol suppresses the expression of proinflammatory mediators in macrophages by inhibiting the activation of the TAK1 and Akt proteins. This, in turn, prevents the activation of NF-kB, which would otherwise produce a variety of different proinflammatory mediators via a cascade effect. In addition, taraxerol has been shown to suppress the activity of acetylcholinesterase (AChE) in a dose-dependent way [49,50]. Taraxerol is a bioactive metabolite found in certain higher plants. It is known to offer a variety of selective biological effects, which are particularly useful in medical contexts. In addition to its antioxidative capabilities, taraxerol possesses other effects, including antidiabetic, anticancer, and anti-inflammatory [51]. Because of these possibilities, taraxerol has the potential to be an innovative and versatile medication. Furthermore, taraxerol has the potential to develop into an effective cardioprotective molecule, which is important from a clinical perspective.

## 4. Materials and Methods

### 4.1. Chemical Reagents

Taraxerol gift sample obtained from Birla Institute of Technology, Mesra, India. Isoproterenol (CAS no: 51-30-9, TCI chemicals Pvt. Ltd., Chennai, Tamil Nadu, India), malondialdehyde colorimetric assay kit, Northwest life sciences (SR Biosystem Pvt. Ltd., New Delhi, India), lactate dehydrogenase colorimetric assay kit and ELISA kit (Abcams, Cambridge, MA, USA), and creatine kinase mono-enzyme B, TNF-α, NF-kB, and IL-6 (Sigma Aldrich, St. Louis, MO, USA). All other reagents were of commercial analytical grade. The commercial animal diet was obtained from a local supplier in Saudi Arabia. 

### 4.2. Animals

In order to conduct this research study, it was necessary to obtain approval from the Standing Committee of Bioethics Research (SCBR) at the College of Pharmacy, Prince Sattam Bin Abdulaziz College in Saudi Arabia. The SCBR carefully reviewed our methodology and approved it under approval number SCBR-037-2022. Male Sprague Dawley rats with an average weight of 240 ± 30 g were used as subjects for the study. To ensure that all rats were healthy prior to the experiments, they were given unrestricted access to food and water for a period of time before being randomly divided into five groups of six rats each, resulting in a total sample size of thirty animals. Isoproterenol—known for its ability to induce experimental myocardial infarction—was dissolved in normal saline and injected subcutaneously into the above rats for two consecutive days. In this way, we were able to collect data on how these injections affected cardiac function in subsequent studies [52]. Taraxerol, a pharmacological agent of great interest for its potential therapeutic properties, is an insoluble, white-colored solid drug. To overcome this problem and unlock its medicinal benefits, we used a 1% Tween 80 solution as a solvent. This approach has proven effective in improving the solubility of taraxerol and facilitating its efficient delivery in biological systems.

### 4.3. Experimental Protocols

**Scheme 1:**
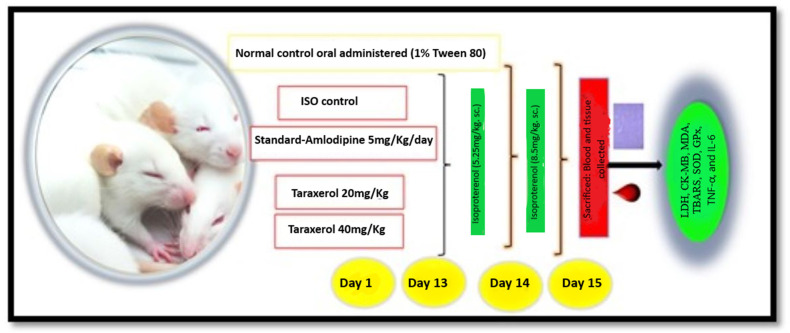
Experimental study design for the animal study.

The experimental protocol for the animal study is given in Scheme 1. Group I was normal control, administered 1% Tween 80 daily. Group II to V, on the 13th and 14th day, received 5.25 and 8.5 mg/kg isoproterenol subcutaneously. Group III was pretreated with amlodipine (5 mg/kg per oral). Groups IV to V were pretreated with oral taraxerol for 15 days (20 and 40 mg/kg, respectively). Any symptomatic changes and mortality in each group are recorded and compared with Group II (rats given isoproterenol alone). After the last administration day, rats fasted overnight with free access to water [52].

On day 15 of the study, all rats in the group underwent noninvasive measurement of hemodynamic parameters. Specifically, heart rate and systolic blood pressure (SBP) were recorded using a rat tail-cuff plethysmography technique and an attached pressure gauge. This procedure was consistent with previous descriptions in the literature and was intended to provide a thorough assessment of cardiovascular function in animal models used for research purposes [50].

On day 15, a comprehensive evaluation of serum cardiac markers was performed by blood collection via retro-orbital puncture. Serum was carefully separated and analyzed for specific oxidative stress markers, including superoxide dismutase (SOD) and glutathione peroxidase (GPx), using advanced UV spectrophotometric techniques. In addition, malondialdehyde levels were determined using an advanced colorimetric assay kit that accurately measured serum MDA concentrations. To gain further insight into the cardiovascular health status of the rats studied, CK-MB and LDH were accurately determined using enzyme-linked immunosorbent assays (ELISA). Following these studies, we sacrificed the rats to perform an autopsy in which their hearts were surgically removed and carefully weighed to obtain accurate measurements.

The heart weight index (HWI) was calculated as follows:

HWI  =  heart weight/body weight (mg/g) [53]

The heart tissues, integral components of the complex cardiovascular system, were carefully collected and preserved in a 10% formalin solution. This careful preservation was performed with the express purpose of performing an in-depth histological study to gain insight into the microscopic structure and function of these vital organs. 

### 4.4. Measurement of Inflammatory Cytokines

During myocardial infarction (MI), levels of proinflammatory cytokines, namely TNF-α and IL-6, are often elevated. These cytokines play a critical role in initiating and propagating inflammatory responses that lead to cardiac injury. To accurately measure the concentrations of TNF-α and IL-6 in serum during MI, we used a reliable commercial ELISA kit from Sigma Aldrich in the United States, following recommended instructions. By accurately measuring these important biomarkers, we hope to better understand the pathophysiology underlying the development of MI and potentially find new therapeutic targets for this debilitating disease.

### 4.5. Histology of Cardiac Tissue

In the careful removal of the hearts from the various groups, a delicate removal practice was used aimed at preserving the integrity of each specimen. The precious organs were then carefully enclosed in blocks of paraffin wax, which would provide optimal protection during transportation and handling en route to their final destination—the pathology laboratory. These invaluable specimens were then sent for extensive histological analysis at the pathology laboratory, where a team of qualified professionals will conduct the examinations.

### 4.6. Statistical Analysis

The experimental results were expressed as the means ± standard error mean (n = 6). The results were statistically analyzed by GraphPad Prism 7.0 software, (San Diego, CA, USA) and one-way analysis of variance (ANOVA) followed by Tukey’s multiple tests. The graph was plotted in Origin software.

## 5. Conclusions

The present study addresses the intricate details of the potential of taraxerol in attenuating myocardial necrosis induced by ISO, specifically examining its cardioprotective properties in a rat model. As a result of ISO, through a comprehensive study and analysis of various factors, it was found that pretreatment with taraxerol effectively protected the rat heart from MI-induced damage. This research contributes to our understanding of the beneficial properties of taraxerol and how they can be used for medical purposes to protect against cardiovascular disease. Through our research, we were able to provide compelling experimental evidence supporting the beneficial effects of taraxerol on heart health. When administered at doses of 20 mg/kg and 40 mg/kg, we observed a significant reduction in cardiac biomarkers while simultaneously increasing antioxidant activity, indicating clear benefits for cardiovascular function. Furthermore, a detailed study in experimental rats revealed that taraxerol not only enhanced heart function but also maintained histo-architecture, an essential aspect of healthy tissue growth and development. These results suggest that taraxerol has the potential to serve as a highly effective means of promoting long-term heart health by improving multiple aspects of cardiac well-being simultaneously. The results obtained in these tests are significant and require further investigation to gain a more complete understanding of the action of taraxerol and to elucidate the intricate mechanisms underlying its action. It is imperative to deepen this area of study to fully understand the potential benefits and drawbacks of this compound. Therefore, further research is needed to explore and decipher the complex interactions underlying the physiological effects of taraxerol.

## Figures and Tables

**Table 1:** Effects of different treatments given on antioxidant levels.

Treatment	MDA (nmol/g of Serum)	GPx (Units/mg of Protein)	SOD (Units/mg of Protein)
Normal control	1.05 ± 0.67	4.12 ± 0.27	5.54 ± 0.63
ISO control	5.35 ± 1.07 *	1.13 ± 0.18 *	0.22 ± 0.09 *
Amlodipine	2.50 ± 0.86 #	3.85 ± 0.32 #	4.08 ± 0.82 #
Taraxerol 20 mg/kg	3.28 ± 0.89 *	1.52 ± 0.28 *	1.18 ± 0.52 #
Taraxerol 40 mg/kg	2.88 ± 0.93 #	2.89 ± 0.55 #	2.88 ± 0.75 #

* *p* < 0.01, as compared with normal control; # *p* < 0.01, as compared with ISO control.

## Data Availability

The data presented in this study are available on request from the corresponding author. The data are not publicly available due to our university policies.

## Sample Availability

Samples of the compounds are available from the authors on request.
